# Functional Relation of the Eye and Ear Curiously Shown by a Case of Mixed Astigmatism

**Published:** 1906-12

**Authors:** R. R. Daly

**Affiliations:** Atlanta, Ga.


### FUNCTIONAL RELATION OF THE EYE AND EAR CURIOUSLY SHOWN BY A CASE OF MIXED ASTIGMATISM.*

By R. R. DALY, M.D.,

ATLANTA, GA.

There is nothing new under the sun, but many surprises continue to occur.

The term hallucination, meaning a false sensory perception of central origin, has long been used among alienists to indicate an important symptom of mental disease. The term illusion, meaning a false sensory perception, with an origin in the environment, indicates a less important and more common symptom. The term delusion, meaning the erroneous conception frequently depending upon hallucination and illusion, makes up a trio of words in constant use at asylums and hospitals for the insane, and at the entrance examination of a patient the conditions indicated are sought for as carefully as possible. A man says he knows his enemies are trying to injure him because he hears electric machinery in the wall. He says he hears it then in that particular wall, and the examiner, hearing no sound at all, puts down hallucination of hearing in connection with persecutory delusion and closes the matter for the time. Some similar examples might readily be cited for seeing and the other senses. Indeed, during my time in the hospitals for the insane the case-books bristled with hallucinations.

But are there real hallucinations? Is it possible to have projected into consciousness from the cortex a perception so exactly like one stimulated from the environment as to deceive the patient? We all have vivid memories—"representations and re-representations" of Spencer—but we can distinguish them as such very clearly, though the face of some loved one may be before us and the music of the voice in our ears with startling distinctness.

---

*Read before the Fulton County Medical Society, At'anta, Ga., Nov. 15, 1906.

They are concepts and not percepts, and we know the difference.

Did this man, hearing the electric machinery where apparently there was no sound to suggest it, get the stimulus from inside the skull as a memory and mistake it? Did some overwrought psychomotor cell cause a false projection of the exact noise a machine would make?

Or is there a simpler way of accounting for his idea by tinnitis aurium and other impairments of hearing which he mistakes for the sound of a machine and which, of course, the examiner can not hear?

Might not the girl who saw the devil frequently following her around have astigmatism, asthenopia and diplopia to account for her disordered fancy of seeing him?

These not only might happen, but they did happen and were proved to exist by careful examinations. Not only that, but false percepts disappeared directly the organs were put in order. So, then, really we had illusions instead of hallucinations to deal with and the question arose, were there any real hallucinations? That can not be answered until many cases have been authoritatively examined and proved. It was in a search to establish this thesis, which is not yet completed, that the case to be described was found.

A married woman, about thirty years of age, was admitted to the hospital with a history of gradual breaking in health, severe headaches, periods of depression and excitability which took the form of religious fear or exaltation. She was incoherent and talked of seeing and hearing strange things too varying and indefinite to describe.

According to routine, she was put to bed in a private room for a few days; a trained nurse was in attendance and the doctor frequently at hand to gain her confidence and learn all he could concerning her. The room was pleasant and the light subdued to make her comfortable. The usual arrangements as to selected food, baths, etc., were followed and in a few days she became quiet and began to improve physically. She complained of headaches about the brows and temples, her lids were slightly reddened and she would draw them together in attempting to see. It was ordered that she go to the ophthalmological room as soon as she was strong enough, but meantime I was requested to see her and so became acquainted with her. She talked of seeing strange things before coming to the hospital, and said that oddly enough since her arrival they had taken definite form. Her memory was excellent for all that had occurred. Describing the form, she said that at night she saw the Cross on the wall opposite to the foot of the bed. It was bright, the Savior was upon it, and usually angels came and sang sweetest music before the vision disappeared. It would go when she closed her eyes and sometimes it would reappear when she opened them. It never came in the daytime. Sometimes it depressed her on account of her sins, and at others it made her happy because divine blessing seemed to be near. In either event she said she prayed out loud when she saw it.

There was nothing upon the wall to suggest the vision, nor could anything about the room be made a basis of illusion. Here, then, was an hallucination to hunt down. She was not yet in condition to leave her bed for an examination, so we had for the time to confine our search to the patient's environment. That night we were stationed in the corridor outside her room, just after she was prepared for sleep. Her door was closed and the room was in darkness. She knew a nurse was within call. In a few minutes we heard her gently praying and we entered her room immediately. She knew us instantly and was not annoyed. She said she had seen the Cross and had heard the music, but our coming had dispersed them. In order exactly to reproduce the circumstances under which she had her vision, the door was closed and the room left in darkness except for a circle of bright light about three inches in diameter which appeared on the wall beyond the foot of the bed. It was caused by the light from an electric lamp in the corridor outside coming through a small hole in the heavy cloth with which the transom was screened. Of course it was accidental. Upon being asked, she said she always saw the bright Cross at that spot, and in a moment she said she could see it there then, but could not hear any singing.

The hole was closed and she saw no more crosses. Her health steadily improved and she said nothing of seeing unusual things in the daytime. In a few days her eyes were examined and she discovered mixed astigmatism + .75 — .50 with the rule. Lenses were immediately ordered and she was instructed to wear them all the time. In two weeks she was accustomed to them. Her headaches had disappeared, the margins of the lids were less red, and she was sitting about the grounds. Then one night the hole in the transom was reopened without her knowledge. After a little time we heard her weeping and went in to inquire the cause of her grief. She said the visions had come again and she feared she would never get well. She was told to put on her glasses and try to see the Cross, as she had done before when we were with her. She did so, but the Cross would not appear; nothing but the spot could be seen, much to her surprise and pleasure. The experiment was repeated. Without the lenses she saw a blurred image resembling a Cross; with them, all was in focus, and she was made to understand that the trouble had been in her eyes rather than in her mind.

The physician can readily see that with alternate effort and relaxation of the ciliary body to overcome a mixed astigmatism, first the perpendicular and then the horizontal diameters of the spot might be brighter and suggest the Cross. Her imagination and association supplied the Savior and at first thought the music. But why the music? There was grief at the Crucifixion. Had we a right to leave the music to her association of ideas? Questioning elucidated only that the music came secondary to the vision of the Cross and did not always occur.

After a few days she was examined in other surroundings and with pure empiricism I attempted the experiment of paralyzing her accommodation by concentrated bright light impinging upon the pupil. My patient by this time was very friendly and interested in all we did for her. She had the freedom of the city and was nearly well enough to be discharged. We were delaying only for her weight to be fully regained. She sat there with the light close to her eye, streaming directly into the pupil, when suddenly she turned and asked what bell was ringing so finely. My stop watch, held through force of habit in psychological examinations, showed eighteen seconds. We questioned her about the bell. It was not a street-car or house bell or any ordinary sound, but a fine musical note she had heard. She was asked to disregard it for the moment and return to the experiment in hand, which she readily did. In fifteen seconds she turned, saying she heard the sound again. And after that, with varying degrees of intensity of light, she heard the sound according to the effort she put forth in looking at the orifice in the chimney. We believed and still believe that the angel's music was thus accounted for.

I shall not attempt to indicate the pathway between the ultimate cortical centers of sight and hearing along which this peculiar stimulus traveled. We all know there is a close functional relationship between the two senses. It is a matter of every-day experience.

For a common example, recall the association that occurs whenever one reads to himself. The sound of the words seen is part of the concept. We are taught in the beginning to read out loud and we read in the same way with our brains, whether we use the mouth or not. The whole chain of functional association is so firmly established that even men of great cultivation, accustomed to read silently, are often seen to be moving their lips slightly.

Those of you who have learned to read a foreign language remember how your inability to pronounce the words correctly in your minds retarded the reading, even though the translation or the idea to be conveyed was quickly taken up. The eyes and the ears work constantly together and a mistake in one part of the path of perception may be projected into the next uncorrected. There was a connection in this patient which caused an illusion that seemed at first a pure hallucination. We had comitant illusions of sight and hearing due to strong outside stimuli of the eyes.

We have good reason for never studying either sense entirely independently of the other. We know the functional relation of smell and taste, and we must not forget the inter-relation of all the senses in studying a case of any complexity unless we are willing to lose the whole secret sought for.

This lady fully recovered. She went home possessed of several pairs of accurately adjusted glasses, and has remained in ordinary health for the last five years.

In reviewing her history, one is impressed with the important part the uncorrected error of refraction played in causing the mental disturbance. Indeed, it seemed to be the primal factor, because so soon as it was attended to she became well. But in any event it is very probable that if the eyes had been relieved of strain and the head thus freed from pains as soon as they appeared, her mental trouble would have been prevented. The insane cases do not come to our offices for treatment after the mind has been affected, but those who may become insane do come to us, and it is always worth thinking of, that a high-strung, imaginative, nervous patient may have illusions of sight and consequent mental alienation, if eyestrains are not recognized and completely relieved.

Asylum physicians know that frequent cures occur at their institutions through corrective work on the special sense organs, and the modern institution is not well equipped unless it has all the appliances for the study and treatment of diseases of the special senses. But preventive treatment is better, and that we can give by painstaking, accurate work in our offices. It is true the patient may not know from what danger he has been saved, but that need not detract from the satisfaction of feeling that our work has been well done—better, in fact, than even a grateful patient realizes.

*English-American Building.*

---

IN the presence of a tumor in the midline between umbilicus and pubes, the possibility of a cyst of the urachus must be borne in mind. It may simulate an ovarian cyst or other tumor, or a distended bladder.—*American Journal of Surgery.*

